# Analysis of CDR3 Sequences from T-Cell Receptor β in Acute Respiratory Distress Syndrome

**DOI:** 10.3390/biom13050825

**Published:** 2023-05-12

**Authors:** Sara Hey, Dayjah Whyte, Minh-Chau Hoang, Nick Le, Joseph Natvig, Claire Wingfield, Charles Onyeama, Judie Howrylak, Inimary T. Toby

**Affiliations:** 1 Department of Biology, University of Dallas, Irving, TX 75062, USA; shey@udallas.edu (S.H.);; 2 T.C Pediatrics, Bedford, TX 76021, USA; 3 Pulmonary, Allergy and Critical Care Division, Penn State Milton S. Hershey Medical Center, Hershey, PA 17033, USA; jhowrylak@pennstatehealth.psu.edu

**Keywords:** Complementarity Determining Region 3, immune sequencing, Acute Respiratory Distress Syndrome, T-cell receptor, sequence analysis, biochemical properties and clonality

## Abstract

Acute Respiratory Distress Syndrome (ARDS) is an illness that typically develops in people who are significantly ill or have serious injuries. ARDS is characterized by fluid build-up that occurs in the alveoli. T-cells are implicated as playing a role in the modulation of the aberrant response leading to excessive tissue damage and, eventually, ARDS. Complementarity Determining Region 3 (CDR3) sequences derived from T-cells are key players in the adaptive immune response. This response is governed by an elaborate specificity for distinct molecules and the ability to recognize and vigorously respond to repeated exposures to the same molecules. Most of the diversity in T-cell receptors (TCRs) is contained in the CDR3 regions of the heterodimeric cell-surface receptors. For this study, we employed the novel technology of immune sequencing to assess lung edema fluid. Our goal was to explore the landscape of CDR3 clonal sequences found within these samples. We obtained more than 3615 CDR3 sequences across samples in the study. Our data demonstrate that: (1) CDR3 sequences from lung edema fluid exhibit distinct clonal populations, and (2) CDR3 sequences can be further characterized based on biochemical features. Analysis of these CDR3 sequences offers insight into the CDR3-driven T-cell repertoire of ARDS. These findings represent the first step towards applications of this technology with these types of biological samples in the context of ARDS.

## 1. Introduction

Acute Respiratory Distress Syndrome (ARDS) is an illness that typically develops in people who are significantly ill or have serious injuries. Within a few hours, patients with ARDS will develop severe shortness of breath, which is one of its most common symptoms. ARDS has two pathological phases: (1) the exudative phase (early phase) and (2) fibroproliferative phase. ARDS is characterized by fluid build-up that occurs in the alveoli of the lungs. This exudative process impairs oxygenation. The lack of sufficient oxygen explains why patients with ARDS are placed on supplemental oxygen for milder symptoms, while severe cases are placed in a mechanical ventilation system. ARDS occurs within one week of a known clinical insult or new or worsening respiratory symptoms. It is a consequence of various several risk factors, including direct (e.g., bacterial or viral pneumonia, gastric aspiration, lung contusion, toxic inhalation, and near drowning) or indirect (e.g., sepsis, pancreatitis, severe trauma, massive blood transfusion, and burn) lung injury. ARDS can also develop as a secondary response to a variety of infectious or inflammatory insults to the lung that occur by direct (e.g., pneumonia) or indirect injury (e.g., peritonitis) [1,2,3,4]. Individuals with ARDS have elevated levels of inflammatory mediators, such as TNF-α, IL-1β, IL-6, and IL-8, in lung-lining fluid as well as in the circulation. These individuals also exhibit an accumulation of inflammatory cells and protein-rich exudates in the alveolar spaces [5,6]. Consequently, localized inflammatory processes are detectable during ARDS, with several publications indicating that T-cells contribute to an aberrant response, which leads to excessive tissue damage and, eventually, ARDS. Numerous studies have focused on the use of whole blood samples for the extraction of immune receptors. It has been documented that lavage lymphocytes contain elevated levels of T-cells, and analysis of cells from bronchoalveolar lavage (BAL) fluid may have diagnostic, therapeutic, and investigative value in evaluating individuals with lung disease [7,8]. The risk of death from ARDS increases with age and severity of illness, while those that do survive may experience lasting damage to their lungs. T-cells are key players in the adaptive immune response. This response is governed by an elaborate specificity for distinct molecules and the ability to recognize and vigorously respond vigorously to repeated exposures to the same molecules [9]. The immune repertoire refers to the composite of all T-cells in an individual. Recent advancements have provided us with the tools and capabilities to take a deeper look at the T-cell receptors and examine their core components. 

The immune repertoire refers to the full set of composite genes in an individual at a single point in time. V(D)J recombination is the distinguishing feature of adaptive immunity and enables effective immune responses against an essentially infinite array of antigens. The genomes of T-cells undergo combinatorial shuffling (somatic rearrangement) of cell-surface receptor gene segments, allowing for a finite genome to encode many trillions of possible receptors. Most of the diversity in these T-cell receptors (TCRs) is contained in the Complementary Determining Region 3 (CDR3) regions of the heterodimeric cell-surface receptors [10,11]. For the TCR, the CDR3 regions are formed by rearrangements of (1) variable and joining (VJ) gene segments for the alpha (α) and gamma (γ) chains, and (2) variable, diversity and joining (VDJ) gene segments for the beta (β) and delta (Δ) chains. Most T-cells are αβ (alpha beta), while γΔ (gamma delta) T-cells are much less common and mainly found in the gut microbiota. These genetic recombination mechanisms create a large diversity of clonal TCRs within a healthy person, which is sufficient for one or more adaptive immune cells to bind to almost any antigen and initiate an immune response [12,13,14,15]. In addition to generating a diverse set of antigen receptor molecules, the adaptive immune system functions in part by clonal expansion; in an adult human, there are millions of different TCR rearrangements carried by several billion circulating T-cells [13]. Previous work published by both Fink et al. and Winoto et al. showed that in some cytochrome c-specific T-cell clones, changes limited to the junctional regions of TCR sequences altered the specificity for peptide without altering MHC specificity. In addition, the finding that some cytochrome c-specific TCRs show selection for certain amino acids with the CDR3-equivalent region suggests that these residues are important for peptide recognition [16,17]. These highly variable CDR3 sequences are important for the recognition of an antigen on the HLA molecule [18]. Results from previous immune sequencing experiments demonstrated that specific TCRβ receptor sequences could be tracked and that CDR3 sequences from these receptors could be extracted for further analysis. It is not clear whether CDR3 sequences derived from ARDS-affected individuals will convey a high percentage of sequence similarity and variations in biochemical features as compared with those derived from non-ARDS-affected individuals.

## 2. Materials and Methods

### 2.1. Sample Collection, Immune Sequencing, and Retrieval of CDR3 Sequences

Lung edema fluid from 4 individuals diagnosed with ARDS (2 males and 2 females) and 3 individuals without ARDS (2 females and 1 male) were retrieved and utilized as the input biological materials for the extraction of genomic DNA. ARDS samples were from individuals diagnosed with ARDS using the Berlin criteria. Non-ARDS samples were from individuals who had either sepsis or SIRS, resulting in one or more major organ systems’ failure and necessitating a stay in the intensive care unit (ICU). All ethical protocols for the use of human tissue for research were adhered to in accordance with IRB approval. Samples were obtained using IRB approval (IRB00007703) and material transfer agreement (HY-2102-22M, Toby I- UDallas and Howrylak J- Penn State). Genomic DNA (gDNA) from each sample was extracted using a Qiagen miniprep genomic DNA kit [19]. gDNA from each sample were submitted to Adaptive Biotechnology for implementation of their ImmunoSeq assay protocol for extraction of TCRβ receptors using the human TCRβ assay. Adaptive Biotechnologies is the gold standard for accurate, quantitative human TCRβ sequencing and has established technology for the extraction of TCRβ. Their immune sequencing solution provides quantitative insight into the breadth and depth of the adaptive immune repertoire by controlling for PCR amplification bias with a combination of highly optimized primers, a set of synthetic immune molecules as built-in controls, and advanced bioinformatics [20]. We have previously utilized this approach for studies contributing to the design of a community resource of data from immune sequencing platforms [8,11]. The ImmunoSeq assay rapidly performs highly accurate T-cell sequencing followed by integrated machine learning analysis using the ImmunoSeq analyzer, an advanced bioinformatics software that is used to synthesize the data from the assay. From each sequenced sample gDNA, the output includes template quantitation, cell counts, and accurate repertoire metrics. CDR3 sequences were further extracted from the processed data and further analyzed using external software tools.

### 2.2. Protein Isoelectric Point

CDR3 nucleotide sequences were parsed out based on sample groupings and extracted into individual sequence files. Data were then extracted from each of the sample files and imported into the Sequence Manipulation Suite: translate [21]. The first step of the analysis was to convert them into protein sequences. The corresponding protein sequences were then saved and exported for additional assessments. Protein sequences were next processed for protein isoelectric point analysis using the Sequence Manipulation Suite: Protein Isoelectric Point function. The software calculated the theoretical pl (isoelectric point) for each of the protein sequences. The calculated pH across each residue sequence from our study was obtained. This process was replicated for each sample across all CDR3 residues. Once the process was completed, the data were further compiled, and the use of scripting to enable sample sorting was performed.

### 2.3. Immunogenicity Analysis

CDR3 sequences were extracted and then placed into separate output files per sample. Each sample was stored in a FASTA file. Each FASTA file was then imported into the Immune Epitope Database and Analysis Resource (IEDB) for analysis projects. T-cell immunogenicity prediction was performed using the T Cell Epitope Prediction Tool (V2.26), T-cell class I pMHC immunogenicity predictor tool, available from the IEDB resource portal (www.iedb.org, accessed on 22 August 2022) [22]. Default prediction values were utilized for each analysis set. Class I immunogenicity scores were assigned based on the full length of k-mers occurring across all the CDR3 sequences. Each CDR3 sequence was individually scored and reported.

### 2.4. Protein Residue Statistics

CDR3 sequences from all samples were imported into the software for computation of residue statistics using the “Protein Statistics function” [21]. The occurrence of the amino acid residues was calculated and summarized based on amino acid properties. The groupings were based on the International Union of Pure and Applied Chemistry-International Union of Biochemistry nomenclature. The amino acid properties were categorized as follows: (1) Aliphatic-G,A,V,L,I; (2) Aromatic F,W,Y; (3) Sulphur C,M; (4) Basic K,R,H; (5) Acidic B,D,E,N,Q,Z; (6) Aliphatic hydroxyl S,T; (7) tRNA synthetase class I Z,E,Q,R,C,M,V,I,L,Y,W; and (8) tRNA synthetase class II B,G,A,P,S,T,H,D,N,K,F.

### 2.5. CDR3 Clonality

**Figure 1:**
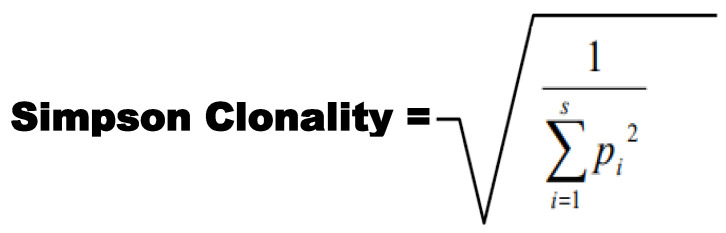
Simpson Clonality equation utilized for the calculation of clonality.

Clonality was taken as a measurement of the sample evenness, not the evenness of the underlying pool from which the sample was taken. Here, clonality was used to compare samples with different amounts of input material. Simpson Clonality was the metric utilized for comparing sequence repertoires. Simpson Clonality values range from 0 to 1, where values approaching: 0 represent a completely even sample, and 1 represents a monoclonal sample. Simpson Clonality is the square root of Simpson’s Index. Simpson’s Index is 1—Simpson’s Diversity Index [23,24,25,26,27,28,29,30,31,32,33,34,35,36,37,38,39,40]. The ImmunoSeq analyzer software was utilized to compute the Clonality score for samples, and the associated equation is shown in Figure 1.

### 2.6. Statistical Analysis

CDR3 sequence analysis was performed using the ImmunoSeq analyzer software. The calculations for length were performed using the sequenced length of the CDR3 nucleotides, starting from the first base of the codon for the conserved cysteine in the V gene through the last base of the codon for the conserved residue in the J gene that ends the CDR3 [19]. Further analysis of the CDR3 residues was performed using Dunn’s test, ggplot2, and hist packages in R [41]. Multiple sequence alignment was performed using the ClustalOmega software [42,43].

## 3. Results

### 3.1. Sequence Analysis

#### 3.1.1. CDR3 Sequences Have Varying Length Distributions

**Figure 2:**
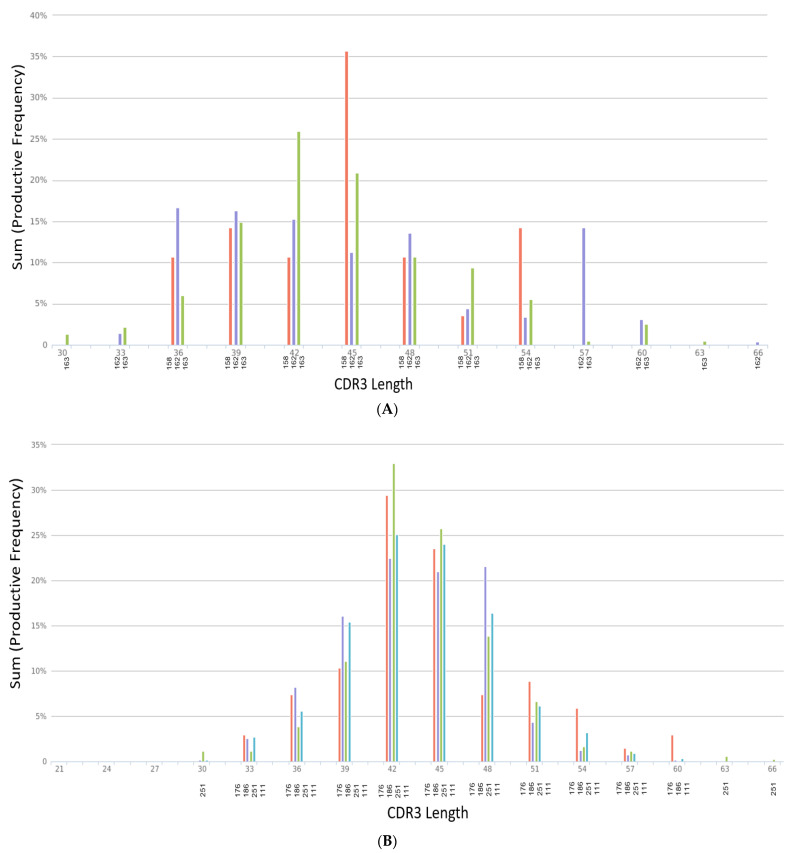
(**A**) CDR3 length distribution across non-ARDS samples. (**B**) CDR3 length distribution across ARDS samples.

The length of the CDR3 in nucleotides was assessed (Figure 2A,B), starting from the first base of the codon for the conserved cysteine in the V gene through the last base of the codon for the conserved residue in the J gene that ends the CDR3. Analysis of the CDR3 length from each sample group shows that the sequence length for ARDS samples ranges from the smallest length at 21 to the largest length at 69 (Figure 2A). The non-ARDS samples range in length from 30 to 66 (Figure 2B). The percent frequency of productive sequences found at a given length across all samples averages ~36%. Summary statistics from each sample grouping show the following: The mean length for non-ARDS samples = 47 amino acids, with variance = 92.23, kurtosis = −0.83, and skewness = 0.16. The mean length for ARDS samples = 39 amino acids, with variance = 139.76, kurtosis = −0.02, and skewness = −0.54.

Figure 2A’s CDR3 length distribution is across non-ARDS samples. The X-axis denotes the length of nucleotides from 30 to 66. Samples 158, 162 and 163 are denoted on the X-axis labels as red, purple and green bars respectively. The Y-axis displays the sum productive frequency for the residues of the indicated length from a given sample. This frequency is reported as a percentage of the filtered sequences.

In Figure 2B, the X-axis denotes the length of nucleotides from 21 to 69. Samples 176, 186, 251 and 111 are denoted on the X-axis labels as red, purple, green and blue bars respectively. The Y-axis displays the sum productive frequency for the residues of the indicated length from a given sample. This frequency is reported as a percentage of the filtered sequences.

#### 3.1.2. Immunogenicity Ratio of CDR3 Sequences

Immunogenicity scores (Table 1) were retrieved from each of the CDR3 sequences from each sample. For the ARDS samples, the percentage of CDR3 sequences with positive immunogenicity scores ranges from ~32–34%. For the non-ARDS samples, the percentage is slightly lower in the range of ~28–30%. The difference in the variance of immunogenicity scores between the ARDS samples and non-ARDS samples is statistically significant (*p* < 0.05).

### 3.2. Biophysiochemical Properties

#### 3.2.1. CDR3 Residues Have Variations in Isoelectric Point

The isoelectric point (Supplemental Table S1) was obtained from each CDR3 residue found in each of the samples. The mean recorded pH for the non-ARDS samples is 5.73, and the mean recorded pH from the ARDS samples is 5.43. The range of pHs from the non-ARDS samples is from 3.23 to 12.05. The range of pHs for CDR3 residues from the ARDS samples was from 3.17 to 12.98. For the non-ARDS samples, the percentage of CDR3 residues with reported isoelectric points of <7 is 76%, 75%, and 69%. For the ARDS samples, the percentage of CDR3 residues with reported isoelectric points of <7 is 76%, 78%, 77%, and 79%.

#### 3.2.2. CDR3 Protein Residue Biochemical Properties

Biochemical properties were analyzed for all CDR3 residues across all samples (Table 2, Supplemental Table S2). The tRNA synthetase class I and tRNA synthetase class II residues represented the largest percentage of properties distributed across the residues from ARDS samples and non-ARDS samples. The composition of the ARDS and non-ARDS CDR3 residues consists of 36% and 64% for the tRNA synthetase class I and class II, respectively. Aliphatic hydroxyl (S,T) and Aliphatic (G,A,V,L,I) residues consist of ~50% composition for the ARDS samples and 49% composition for the non-ARDS samples. Aromatic (F,W,Y) residues consist of 16.4% for the ARDS samples and 15.87% for the non-ARDS samples. Sulphur (C,M) residues consist of 7.22% for the ARDS and non-ARDS samples. Basic (K,R,H) residues consist of 5.94% for the ARDS samples and 6.96% for the non-ARDS samples. Acidic (B,D,E,N,Q,Z) residues consist of 17.17% for the ARDS samples and 17% for the non-ARDS samples.

### 3.3. Clonality

#### CDR3 Clonality and Shared Sequences

**Figure 3:**
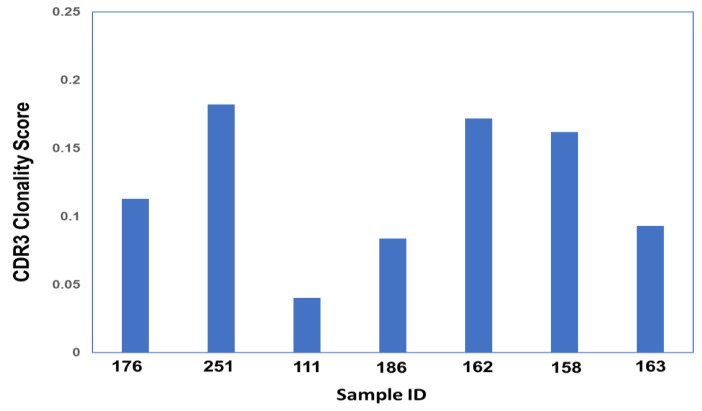
CDR3 Simpson Clonality Score across all Samples.

**Figure 4:**
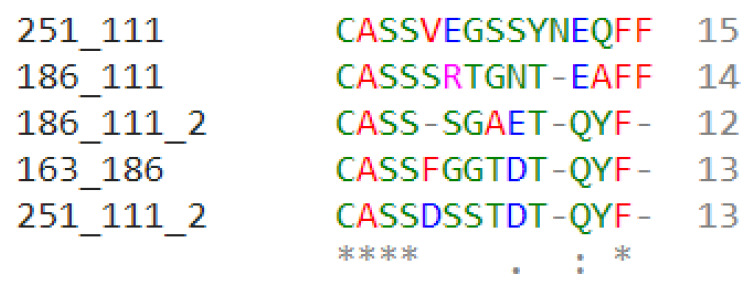
Multiple Sequence Alignment (MSA) of shared CDR3 sequences.

Productive Simpson Clonality calculations (Figure 3, Supplemental Table S3) were obtained for each sample in the study. The scores yield the following: ARDS samples—0.095, 0.144, 0.0406, and 0.2336, and non-ARDS samples—0.1951, 0.2082, and 0.111. The shared sequences found within all CDR3 sample groupings comprise five sequences. There are four sequences shared within three out of the four ARDS samples, and one sequence is shared between the ARDS sample and the non-ARDS sample. Multiple sequence alignment (Figure 4) of the shared sequences show five positions denoted as “*” (positions 1–4 and 14) with a single, fully conserved residue. One position denoted as “:” (position 12) is observed, which indicates conservation between groups of strongly similar properties—roughly equivalent to scoring > 0.5 in the Gonnet PAM 250 matrix. One position denoted as “.” (position 9) was observed, which indicated conservation between groups of weakly similar properties—roughly equivalent to scoring ≤0.5 and >0 in the Gonnet PAM 250 matrix. Sequencing templates obtained from ARDS samples are higher than those obtained from non-ARDS samples. Sample 251 (ARDS sample) is found to be statistically significant in the frequency of clonal sequences as compared with all others (*p* = 0.0248, Dunn’s test). This is based on the max frequency of clonal groups observed (Supplemental Table S3). For CDR3 sequences with clonal groups ≥ 10, positive immunogenicity scores are obtained for 18 individual sequences from the ARDS samples and two sequences from the non-ARDS samples (Table 1).

Figure 3’s Clonality score was obtained for each sample using Simpson’s clonality equation. Samples 176, 251, 111, and 186 correspond to ARDS samples. Samples 162, 158, and 163 correspond to non-ARDS samples.

Figure 4’s MSA above shows the five shared CDR3 sequences within the samples in this study. Samples denoted in this figure as 251, 111, and 186 correspond to the ARDS samples. The sample denoted as 163 corresponds to the non-ARDS sample.

Each sequence is shown with two labels of sample name to correspond to its shared sample pair. Sample numbers after the underscore denote the shared sample. 

An * (asterisk) indicates the positions that have a single, fully conserved residue. 

A “:” (colon) indicates conservation between groups of strongly similar properties roughly equivalent to scoring >0.5 in the Gonnet PAM 250 matrix. A “.” (period) indicates conservation between groups of weakly similar properties, roughly equivalent to scoring ≤0.5 and >0 in the Gonnet PAM 250 matrix.

## 4. Discussion

CDR3 sequences, which are the core component of the T-cell repertoire, contain a remarkable amount of information that provides a snapshot into the active process of molecular interactions during both active disease and in its natural state. This physiological environment has been shown to be altered during periods of illness or compromised health status as compared with a healthy individual. The use of biochemical features from the CDR3 residues represents a novel opportunity to further differentiate the landscape of ARDS-affected individuals from those with other related lung pathologies. The complexity of the biological processes during active diseases makes it often challenging to distinguish ARDS from other lung injury pathologies. Thus, the prospect of a viable biological feature that can further be scrutinized for clinical decision-making would be a benefit. This study takes a first step towards the classification of the biochemical features from CDR3 sequences across the sample groups. Of note, there were considerably fewer sequences obtained from each of the non-ARDS individuals as compared with those with ARDS. An important consideration is that for this study, the non-ARDS individuals were not health controls but rather individuals with other related lung injury pathologies. An assessment of the starting concentration for the input material (genomic DNA) did not suggest that factor as being of significance to the experimental outcome. More studies would be needed to better understand if the differences in receptor sequences obtained from the sequencing experiments are due to specific biological processes or experimental nuances such as collection time, etc. Previous studies have shown that experimental variations may occur in part due to active biological processes and other factors unrelated to the experimental sequencing process. Additionally, the sequencing process in the study utilizes a series of bias-controlled steps to address sampling variations and further utilizes a system of high throughput computing of templates pre- and post-sequencing steps [19].

Previous work has shown that the analysis of CDR3 length distribution is a useful strategy for predictive methods either on its own or coupled with other types of data. The advantage of this method is that the quantitative nature of the analysis can be correlated with the quantitative usage of the corresponding gene. This will also enable the interpretation of the T-cell repertoire in order to provide a snapshot of the immune landscape [44,45,46,47,48]. The approach utilized in this study controls for the degree of repertoire bias encountered in immune sequence data. It also accounts for the individual clonal populations represented by the CDR3 sequences. The isoelectric point of an amino acid is the point at which the amino acid has no net electrical charge. It is an important characteristic of any amino acid because every amino acid has at least two acid–base (titratable) groups. Substitutions in amino acids may impact the stability of the binding activities that ensue [49,50,51]. An analysis of the isoelectric point computed for each of the CDR3 residues from our study did not show any statistically significant differences. However, residue substitutions were observed in the MSA analysis. A structural analysis would be needed to address the impacts of these residue substitutions.

In an adult human, there are millions of different TCR rearrangements carried by several billion circulating T-cells [52]. Characterizing immune repertoires is critical in both basic research and clinical contexts. Previous work has shown that lavage lymphocytes contain elevated levels of T-cells [53,54]. These studies suggest that the analysis of cells from lung fluid may be of diagnostic, therapeutic, and investigative value in evaluating individuals with lung disease [52,53,54]. Immunogenicity can be measured directly in peptide-immunization experiments, as other factors like the right processing of a peptide or the expression of a source protein are excluded from negatively influencing the T-cell response [22]. The software utilized in the study was based on the algorithm developed for immunogenicity predictions. In the previously described development work, the authors first collected a set of immunogenic and non-immunogenic residues and compared the amino acid frequencies in both sets. Their analysis demonstrated that T-cell receptor sequences have strong preferences for certain amino acids, especially Aromatic and large residues. Next, they analyzed the importance of the different positions of the presented peptides with respect to immunogenicity. As expected, the middle part of the presented peptide (P4–P6) was shown to be the most important. These results were validated by combining them into a simple enrichment model and testing if this model could estimate the immunogenicity of new residues [55,56].

To assess the ability of the CDR3 sequences derived from our study to elicit an immune response, we utilized the previously described algorithm based on the underlying premise that certain amino acids are more likely to interact with TCRs and therefore increase the immunogenicity of peptide–MHC–I complexes (pMHCs). We applied the same analysis criteria and parameters to both sample groups, which comprised ARDS-affected individuals and non-ARDS individuals. This approach represents a novel opportunity for us to apply a validated algorithm to uncover insights within CDR3 sequences that may not be obvious from other types of analysis. We found that the overall frequency of positive scoring CDR3 residues was higher in the ARDS samples as compared to the non-ARDS samples. This may, in part, be due to higher clonal sequences being obtained from the ARDS samples. More sampling would be needed to interrogate other potential explanations further. Additionally, we employed techniques for the assessment of protein residue based on biochemical properties. The isoelectric point of an amino acid is the point at which the amino acid has no net electrical charge. It is an important characteristic of any amino acid because every amino acid has at least two acid–base (titratable) groups. Similar to previously published work, we observed that a portion of the CDR3 sequences comprises the Aromatic and Aliphatic groups of residues [57]. There are currently very limited amounts of information on the biochemical properties of CDR3 sequences from immune sequence datasets using lung fluid in the context of ARDS or lung injury; thus, this study contributes sequence datasets and the framework for further exploration into this area of research. Our future work would include the retrieval of more CDR3 sequences to build a library of information from lung fluid-specific TCRβ immune sequence data. Altogether, our additional preliminary data emphasizes the importance and significance of analyzing CDR3 sequences from immune sequence data to characterize the T-cell repertoire.

## Figures and Tables

**Table 1:** Summary of Immunogenicity Score obtained from CDR3 sequences.

Sample ID	Positive Scoring Sequences	Total Number of Sequences	Percent % Immunogenicity Observed	Clinical Diagnosis
111	515	1560	33.01%	ARDS
176	19	56	33.93%	ARDS
186	501	1485	33.74%	ARDS
251	64	202	31.68%	ARDS
158	7	25	28.00%	Non-ARDS
162	33	118	27.97%	Non-ARDS
163	51	169	30.18%	Non-ARDS

**Table 2:** Biochemical properties of CDR3 residues.

Non-ARDS CDR3 Residues	Times Found	Percentage	ARDS CDR3 Residues	Times Found	Percentage
Aliphatic G,A,V,L,I	1270	27.46	Aliphatic G,A,V,L,I	13,491	28.15
Aromatic F,W,Y	734	15.87	Aromatic F,W,Y	7858	16.4
Sulphur C,M	334	7.22	Sulphur C,M	3461	7.22
Basic K,R,H	322	6.96	Basic K,R,H	2846	5.94
Acidic B,D,E,N,Q,Z	786	16.99	Acidic B,D,E,N,Q,Z	8228	17.17
Aliphatic hydroxyl S,T	1005	21.73	Aliphatic hydroxyl S,T	10,611	22.14
tRNA synthetase class I Z,E,Q,R,C,M,V,I,L,Y,W	1668	36.06	tRNA synthetase class I Z,E,Q,R,C,M,V,I,L,Y,W	17,360	36.22
tRNA synthetase class II B,G,A,P,S,T,H,D,N,K,F	2957	63.94	tRNA synthetase class II B,G,A,P,S,T,H,D,N,K,F	30,566	63.78

## Data Availability

Datasets from this study will be made publicly available via NCBI GenBank upon publication. Prior to publication release, study datasets will be archived in Zenodo and can be accessed by submitting a request via email to the corresponding author.

## Supplementary Materials

The following supporting information can be downloaded at: https://www.mdpi.com/article/10.3390/biom13050825/s1, Table S1, Isoelectric point for CDR3 residues found in each of the study samples. Table S2, Biochemical properties for CDR3 residues from all study samples. Table S3, Productive Simpson Clonality scores and template frequency from all study samples.
